# Low But Recoverable Markers of Humoral Immune Response to BNT162b2 in Elderly LTCF Residents Five to Seven Months After Two-Dose Vaccination

**DOI:** 10.3389/fragi.2022.883724

**Published:** 2022-04-25

**Authors:** Marla Delbrück, Sebastian Hoehl, Tuna Toptan, Barbara Schenk, Katharina Grikscheit, Melinda Metzler, Eva Herrmann, Sandra Ciesek

**Affiliations:** ^1^ Institute of Medical Virology, Goethe University Frankfurt, Frankfurt, Germany; ^2^ Institute of Biostatistics and Mathematical Modeling, Goethe University Frankfurt, Frankfurt, Germany; ^3^ Fraunhofer Institute for Molecular Biology and Applied Ecology (IME), Branch Translational Medicine and Pharmacology, Frankfurt, Germany; ^4^ German Center for Infection Research, DZIF, External Partner Site Frankfurt, Braunschweig, Germany

**Keywords:** SARS-CoV-2, COVID-19, immunosenescence, vaccination, immunity, multimorbidity, elderly

## Abstract

The immune response is known to wane after vaccination with BNT162b2, but the role of age, morbidity and body composition is not well understood. We conducted a cross-sectional study in long-term care facilities (LTCFs) for the elderly. All study participants had completed two-dose vaccination with BNT162b2 five to 7 months before sample collection. In 298 residents (median age 86 years, range 75–101), anti-SARS-CoV-2 rector binding IgG antibody (anti-RBD-IgG) concentrations were low and inversely correlated with age (mean 51.60 BAU/ml). We compared the results to Health Care Workers (HCW) aged 18–70 years (*n* = 114, median age: 53 years), who had a higher mean anti-RBD-IgG concentration of 156.99 BAU/ml. Neutralization against the Delta variant was low in both groups (9.5% in LTCF residents and 31.6% in HCWs). The Charlson Comorbidity Index was inversely correlated with anti-RBD-IgG, but not the body mass index (BMI). A control group of 14 LTCF residents with known breakthrough infection had significant higher antibody concentrations (mean 3,199.65 BAU/ml), and 85.7% had detectable neutralization against the Delta variant. Our results demonstrate low but recoverable markers of immunity in LTCF residents five to 7 months after vaccination.

## Introduction

Advanced age is a strong risk factor for severe and fatal disease when infected with SARS-CoV-2 (Figliozzi et al., 2020). In the elderly, coronavirus disease of 2019 (COVID-19) is less commonly asymptomatic (Sah et al., 2021), and respiratory failure and organ dysfunction are common in hospitalized patients (Liu et al., 2020; Neumann-Podczaska et al., 2020). Residency in long-term care facilities (LTCFs) further increased COVID-19-related mortality in the elderly (Amore et al., 2021). Therefore, older adults were designated a high priority group in the roll-out of COVID-19 vaccines (Organization, 2020). In Europe, 30–60% of all COVID-19 deaths were attributed to residents of LTCFs in the early pandemic (Team et al., 2020). First COVID-19 vaccine studies found high efficacy in adults (Baden et al., 2020; Polack et al., 2020), but the elderly, especially with comorbidities and frailty, were commonly underrepresented or excluded (Soiza et al., 2020). Initial COVID-19 vaccine efficacy data were favourable, but efficacy against hospitalization and death was lower in those 80 years of age and older when compared to 65–79 years (Nunes et al., 2021). Accelerated waning immunity due to immunosenescence is concerning, since clinical efficacy for COVID-19 mRNA vaccines has been documented to decrease after vaccinations throughout different age-groups (Goldberg et al., 2021), and may increase with age (Tober-Lau et al., 2021) and other factors.

## Materials and Methods

The goal of this cross-sectional and observatory study was to determine the influence of age, multimorbidity, and body composition on the humoral immune response in elderly residents of LTCFs, five to 7 months after vaccination with BNT162b2.

We formed and compared three groups of study participants, all of whom had completed a two-dose regimen with BNT162b2 four to 6 months before sample collection. Group one consisted of residents of LTCFs who had no evidence of prior infection or breakthrough infection with SARS-CoV-2, and who were at least 75 years of age. Group two was a control group of health care workers (HCWs) from LTCFs, who also had no evidence of prior infection or breakthrough infection with SARS-CoV-2. Inclusion criteria included an age of 18–70 years. Group three comprised LTCF residents who were 75 years of age and older, who had had a PCR-confirmed breakthrough infection no earlier than 14 days after the second vaccine dose.

Written informed consent was obtained from all study participants. The study protocol has been approved by the ethics board of the University Hospital Frankfurt (No. 20–864) and has been registered on the German Clinical Trial Register (DRKS00025813).

Items were recorded in an interview with a study participant or derived from a patient history form with consent of the patient, or legal guardian, if one had been appointed. Study participants from the HCW group provided information on the items themselves. These items were: 1) date of birth 2) current height and weight 3) known medical conditions 4) current medication.

Serum samples were also tested for the presence of anti-SARS-CoV-2 nucleocapsid IgG antibodies to exclude unknown prior infections with SARS-CoV-2. We used the Abbott ARCHITECT SARS-CoV-2 IgG test (Abbott Laboratories. Abbott Park, Illinois, United States). Detected antibodies may be elicited after infection, but not after vaccination with an mRNA vaccine.

Serum samples were tested for the presence of anti-SARS-CoV-2 Spike Receptor Binding Domain (RBD) IgG antibodies. For this, we used the AdviseDx SARS-CoV-2 IgG II assay on the Abbott Alinity i^®^ platform (Abbott Laboratories, Abbott Park, Illinois, United States . The results are provided in standardized binding antibody units (BAU). Detectable antibodies may be elicited after both infection and vaccination with an mRNA vaccine. Results of 0–7.0 BAU/ml were considered negative, 7.1 to < 8.52 BAU/ml were considered borderline, and 8.52 BAU/ml and higher were considered positive.

Serum samples were further analyzed for the presence of antibodies with neutralizing capacity against the Delta variant of SARS-CoV-2, also termed B.1.617.2. Caco2 cells (DSMZ, Braunschweig, Germany, no: ACC 169) were cultured in Minimum Essential Medium (MEM) supplemented with 10% fetal calf serum (FCS), 4 mM l-glutamine, 100 IU/ml of penicillin, and 100 μg/ml of streptomycin at 37 °C and 5% CO2. All culture reagents were purchased from Sigma (St. Louis, MO, United States). For the experimental set up of the neutralization assay FCS supplementation of the culture medium was reduced to 1%. Serum samples were diluted 1:10 and thereafter serially diluted (1:2) and incubated with 4000 TCID50/mL of the Delta variant of SARS-CoV-2 (B.1.617.2) for 1 hour prior to infection of CaCo-2 cells. After 48 h inoculation infected cells were examined for cytopathic effect (CPE) formation by light microscopy to determine the neutralization titer. A titer of 1:10 was considered borderline positive.

To assess comorbidities for each study participant, the Charlson Comorbidity Index (CCI) was calculated. The CCI is a prospectively applicable method for classifying comorbid conditions which might alter the risk of mortality (Charlson et al., 1987), and is widely used to predict long-term survival. This index is based on the number and seriousness of comorbid diseases.

The Drug Derived Complexity Index (DDCI) was calculated using the medical records of each study participant. The DDCI was developed on prescription patterns indicative of chronic disease.

Data analysis was performed using RStudio^©^ version 1.4.1717. The Wilcoxon-Mann-Whitney-Test was used to test homogeneity of age, sex, BMI, time interval between second vaccination and blood draw, anti-RBD-IgG antibody titers, CCI, and DDCI by study group. To determine differences of neutraliZation titers between the study groups we performed the Chi-squared- and the exact Fisher-test. Spearman’s rank, Pearson’s product-moment correlation, and Chi-squared-tests were used to find associations between demographic features and anti-RBD-IgG titers as well as therapy with immunosuppressive medications, the CCI and the DDCI. The association between age and anti-RBD-IgG antibody concentration was analyzed using a linear regression model. A logistic binomial regression model was developed to test several variables for association with a positive neutralization assay.

## Results

Samples were collected from July 22nd to September 17th, 2021, in 16 LTCFs in Hesse, Germany.

The total number of study participants recruited to the group of elderly LTCF residents without evidence of a breakthrough infection after vaccination was 298. The median age was 86 years (Table 1). 114 HCWs were recruited to the first control group, the median age was 53 years. A total of 14 LTCF residents were recruited to the second control group, comprised of residents with evidence of a breakthrough infection with SARS-CoV-2 no earlier than 14 days after the second vaccine dose. The median age was 89 years in this group (Table 1).

**TABLE 1:** Cohort characteristics by study group. Wilcoxon rank sum test was used to test for homogeneity between study groups. The *p*-values can be found in column 5 respectively.

	Group 1 (*n* = 298) *SARS-CoV-2 naive residents ≥* 75 *years of age*		Group 2 (*n* = 114) *SARS-CoV-2 naive HCWs 18 to 70 years of age*		Group 3 (*n* = 14) *Residents with breakthrough infection*		*p*-values group 1 Vs Group 2 group 1 Vs Group 3
Age at day of first vaccination [median]	86 years		53 years		89 years		p_1 vs. 2_ < 0.001
	108: 82.0 to 90.75, range: 75 to 101		IQR: 45.25 to 59.75, range: 24 to 70		IQR: 86.25 to 91, range: 82 to 93		p_1 vs. 3_ = 0.11
Sex [% within each group]	212 female (71.1%)		83 female (72.8%)		13 female (92.9%)		p_1 vs. 2_ = 0.74
	86 male (28.9%)		31 male (27.2%)		1 male (7.1%)		p_1 vs. 3_ = 0.08
BMI [median]	25.3 kg/m^2^		26.4 kg/m^2^		23.34 kg/m'		p_1 vs. 2_ = 0.14
	IQR: 22.4 to 28 6, range. 14.9 to 42.9		IQR: 22.5 to 30.2, range: 18.0 to 50.0		IQR: 20.5 to 28.6, range: 17.0 to 32,1		p_1 vs. 3_ = 0.41
Interval between second vaccination and blood draw [median]	6.49 months		6.46 months		7.13 months		p_1 vs. 2_ < 0.001
	range: 5,08 to 7.71		range: 5.51 to 6.95		range: 5.84 to 7.57		
Interval between breakthrough infection and blood draw [median]	*N.A.*		*N.A.*		3.69 months		*N.A.*
					range: 0.66 to 6.55		
Anti-RBD-IgG antibody titer [mean]	74.97 BAU/ml		159.99 BAU/ml		3,199.65 BAU/ml		p_1 vs. 2_ < 0.001, CI: 76.82 to -48.93
	10R: 11.22 to 61.69, range: 0.09 to 3,385.75		10,R: 57.04 to 176.30, range: 5.61 to 2008.16		IQR: 857.65 to 4,601.88, range: 58.73 to 11,350.00		p_1 vs. 3_ < 0.001, CI: 2,706.83 to 884.39
Neutralization assay [titer, mode]	No neutralization	269 (90.6%)	No neutralization	78 (68.4%)	No neutralization	2 (14.3%)	p_1 vs. 2_ < 0.001
	1:10 (borderline)	14 (4.7%)	1:10 (borderline)	16 (14.0%)	1:10 (borderline)	0 (0%)	p_1 vs. 3_ < 0.001
	1:20	5 (1.7%)	1:20	12 (10.5%)	1:20	0 (0%)	
	1:40	3 (1.0%)	1:40	6 (5.3%)	1:40	1 (7.1%)	
	1:80	0 (0%)	1:80	2 (1.8%)	1:80	4 (28.6%)	
	1:150	3 (1.0%)	1:160	0 (0%)	1:160	2 (14.3%)	
	1:320	2 (0.7%)	1:320	0 (0 °A)	1:320	2 (14.3%)	
	1:640	1 (0.3%)	1:640	0 (0%)	1:640	1 (7.1%)	
	1:1,280	0 (0%)	1:1,280	0 (0%)	1:1,280	2 (14.3%)	
Charlson Comorbidity Index (CCI) [median index]	6		1		6		p_1 vs. 2_ < 0.001
	1QR: 5 to 7, range: 3 to 14		IQR: 0 to 2, range: 0 to 5		IQR: 5 to 7.75, range: 4 to 12		p_1 vs. 3_ = 0.039
Drug Derived Complexity Index (DDCI) [median index]	5		0		8		p_1 vs. 2_ < 0.001
	IQR: 2 to 8, range- −2 to 20		IQR: 0 to 1, range: −7 to 6		IQR: 6 to 10.5, range: 0 to 15		p_1 vs. 3_ = 0.002

The detailed make-up of the study participants is depicted in Table 1, the history of medical conditions, medication and the BMI are in Supplementary Table S1 and Supplementary Table S2.

**FIGURE 1:**
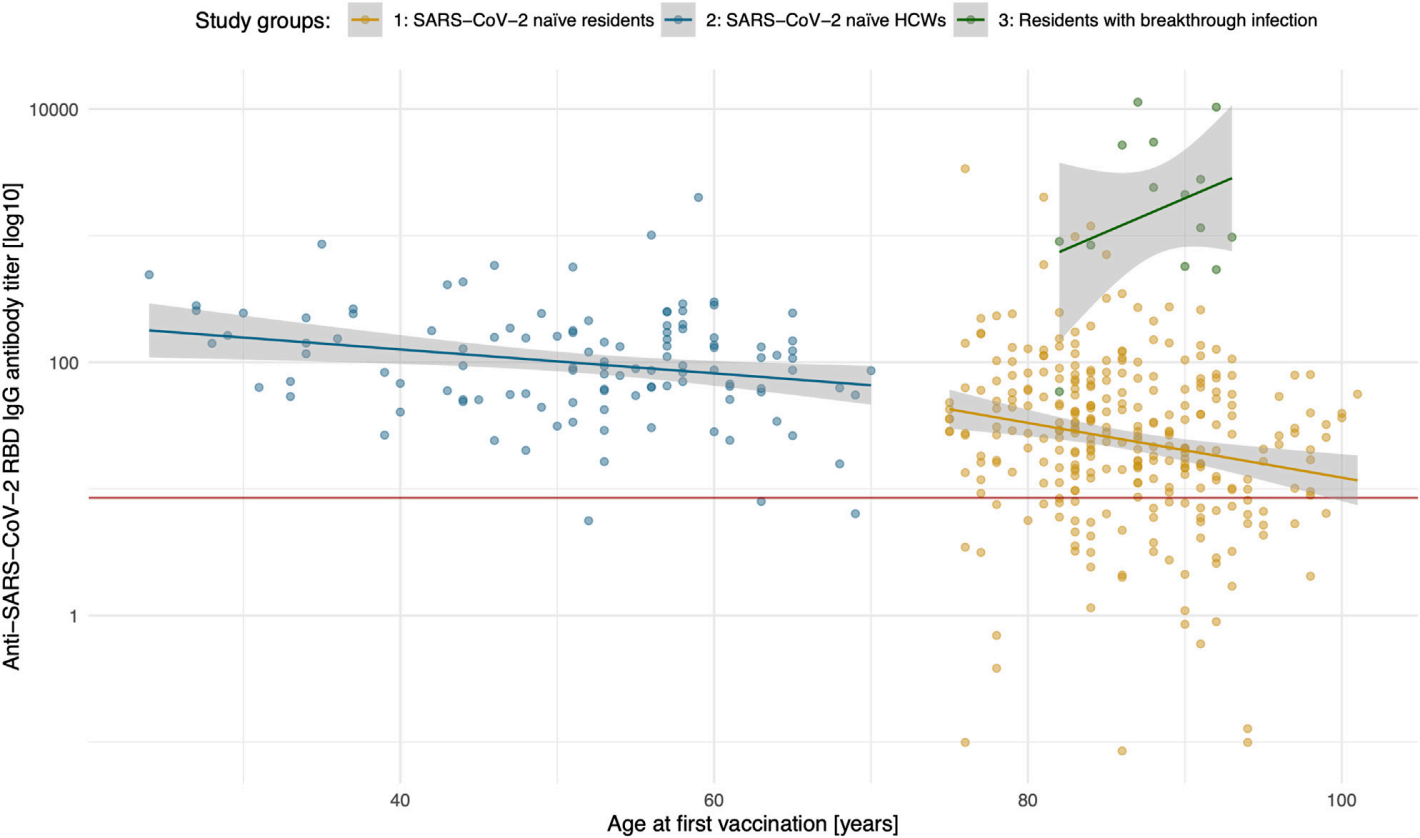
Anti-SARS-CoV-2 RBD IgG titer (logarithmic) by study participants’ age. Group 1 (orange), group 2 (blue), group 3 (green) are depicted. Plotted within is the linear regression (lines in colours of the respective study groups) with the standard error (grey areas, 95% CI). The red line displays the cut-off anti-SARS-CoV-2 spike IgG antibody concentration of 8.52 BAU/ml, which is considered a positive test result (borderline: 7.10–8.51 BAU/ml).

**FIGURE 2:**
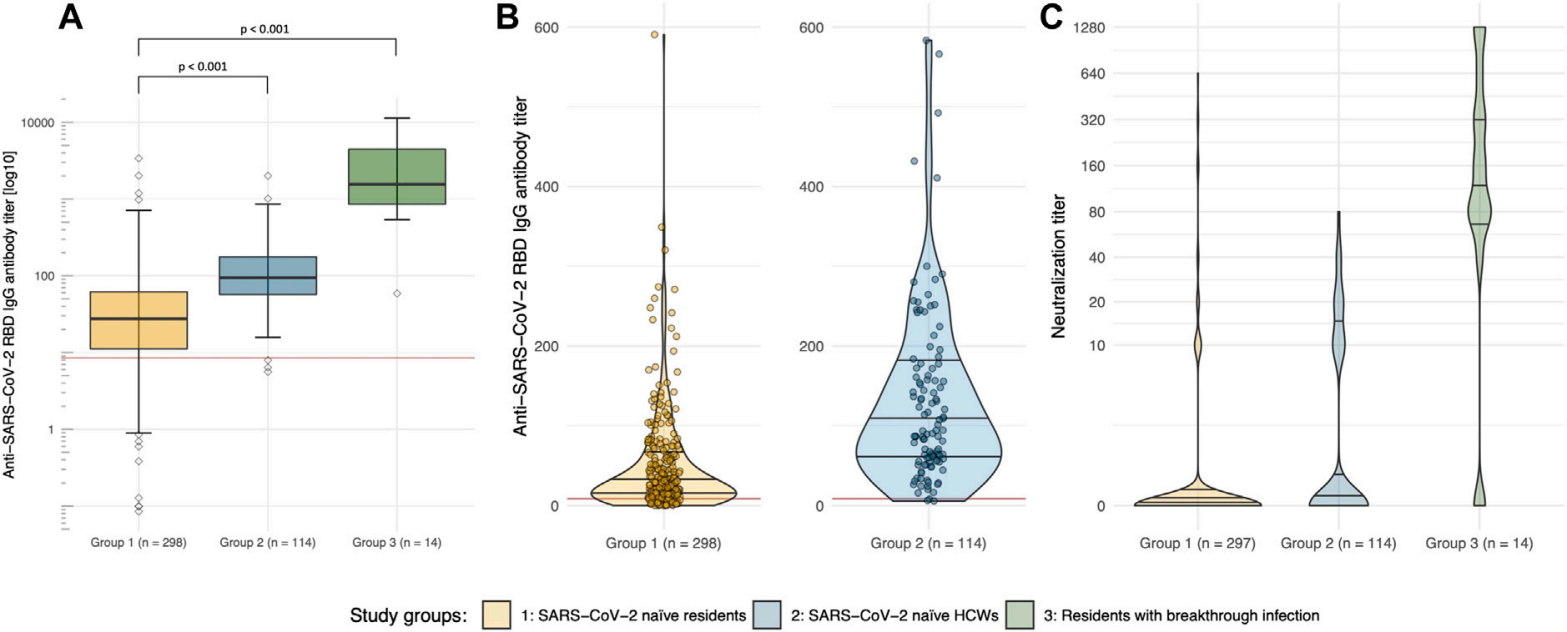
SARS-CoV-2 specific antibody response and neutralization capacity of the Delta variant in elderly LTCF residents. **(A)** Logarithmic depiction in boxplots, 95% CI and IQR (25–75%) of group 1 (residents of LTCFs ≥75 years of age; yellow), group 2 (HCWs at LTCFs, 18–70 years of age; blue), and group 3 (residents of LTCFs after breakthrough infection; green). Rhomboids represent outliers, the red line displays the cut-off anti-SARS-CoV-2 spike IgG antibody concentration of 8.52 BAU/ml, which is considered a positive test result (borderline: 7.10–8.51 BAU/ml). **(B)** Violin plots for visualization of differences in the non-logarithmic distribution of anti-SARS-CoV-2 spike IgG antibody titers of groups 1 (residents of LTCFs ≥75 years of age; orange) and 2 (HCWs at LTCFs, 18–70 years of age; blue). **(C)** Violin plots displaying the different distributions of neutralization titers against the Delta variant between study groups.

We observed a significantly lower IgG antibody concentration targeted at the receptor binding domain (RBD) of SARS-CoV-2 in the elderly LTCF residents when compared to the control group of HCWs (Figures 2A,B). There was a linear decrease of anti-RBD-IgG with age in the elderly, but not in the HCWs of group 2 (Figure 1).

In virus neutralization assay with an authentic Delta variant isolate (Wilhelm et al., 2021), the elderly LTCF residents had lower neutralization against the Delta variant. However, the majority of the HCWs of group 2 also had no detectable neutralization capacity (Figure 2C). We did not observe a significant correlation between time since second vaccine dose and SARS-CoV-2 anti-RBD-IgG or neutralization against Delta, indicating that waning had already occurred at the lower time-point of 4 months.

Antibody testing in the group of LTCF residents after breakthrough infection revealed a higher concentration of anti-RBD-IgG antibodies, as well as higher rates of neutralization of the Delta variant (Figure 1, Figures 2A,C) when compared to all other study participants who had no evidence of an infection with SARS-CoV-2.

The adapted Charlson Comorbidity Index (Ludvigsson et al., 2021), which can be used for risk stratification of COVID-19 mortality (Kuswardhani et al., 2020; Mak et al., 2021), was inversely correlated with SARS-CoV-2 anti-RBD IgG and Delta-neutralization capacity (Table 1). The BMI, however, was not associated with either SARS-CoV-2 anti-RBD IgG or neutralization against the Delta variant.

We furthermore assessed the influence of immunosuppressive medication on the humoral immune response. Only a small number of participants in our study population was prescribed systemic corticosteroids (ATC codes H02AB; prednisolone in varying dosages from 2.5 to 10 mg QD) or other immunosuppressants (ATC codes L04; methotrexate, tacrolimus, dimethyl fumarate, or adalimumab) (Supplementary Table S2). Only 4.4% (13 out of 293) of the elderly, SARS-CoV-2 naïve elderly LTCF residents and 14.3% (2 out of 14) elderly with a breakthrough infection were recorded to be taking corticosteroids, and 0.3% of the elderly as well as 1.8% of HCWs were taking other immunosuppressive medication. We did not observe a significant difference between the anti-RBD-IgG concentration for the small groups of study participants taking these medications, and the other study participants in either one of our three study groups, or with the mean neutralizing titer against the Delta variant.

## Conclusion

In this cross-sectional study we observed low humoral immune markers in the elderly without breakthrough infection, as well as the majority of HCWs. Antibody concentrations of anti-RBD-IgG and neutralizing antibodies were negatively influenced by increasing age. This waning humoral response is recoverable, as observed in the group of elderly with breakthrough infection.

Other studies in the elderly population based on pseudovirus neutralizing assays provided divergently higher rates of neutralization (Collier et al., 2021). This might be due to lower Spike protein density on pseudoviruses compared to authentic viruses. Besides, pseudoviruses are single-cycle viruses, and thus are suboptimal to assess the inherent viral fitness and the impact of non-Spike mutations on the neutralizing sensitivity. Another possible explanation might be that for the neutralisation assays we employed an authentic Delta variant isolate (Wilhelm et al., 2021) rather than a pseudovirus. Clinical isolates harbour additional mutations outside the Spike region which can impact the viral fitness or sensitivity to antibodies in cell culture.

In addition to age, immunogenicity and efficacy of vaccines may be influenced by several factors, including underlying medical conditions. As a strength of our study, we were able to analyze our data using clinical data. We detected a significant reverse correlation of the CCI, which can be used for COVID-19 risk-stratification (Kuswardhani et al., 2020; Mak et al., 2021), with anti-RBD-IgG in the elderly SARS-CoV-2 naïve LTCF residents. This highlights the importance of ensuring a robust vaccine response, especially in the elderly with comorbidities that predict an unfavourable outcome when infected with SARS-CoV-2. Looking at the elderly residents as well as HCWs, the DDCI was significantly correlated with anti-RBD-IgG, but statistical significance was not achieved when analyzing the elderly alone, which may indicate a weak correlation. The validity of the CCI was impaired due to some medical records not providing morbidity in an ICD-10 conformable format, and due to impaired recall in the elderly study participants.

Immunosuppressive medication may impair the humoral immune response. We did not observe significantly lower markers of the humoral response in study participants taking immunosuppressive medication. However, since only a smaller number of participants were on immunosuppressive medication, the validity of these findings is limited.

Body composition with a very low or high BMI may also influence the immune response in the elderly (Mitsunaga et al., 2021). In our study, body composition did not significantly influence the immune response recorded five to 7 months after vaccination. Since a large majority of our study participants were female (Table 1), gender-specific influences mainly affecting males may not have been detected (Yamamoto et al., 2021).

Of note, only 28.6% of the study participants with a documented, PCR-confirmed breakthrough infection had detectable anti-nucleocapsid IgG antibodies. This highlights that infection with SARS-CoV-2 in this group may have occurred without an enduring anti-nucleocapsid response. In line with this, some of the participants among presumably SARS-CoV-2 naïve LTCF residents, could have been infected, despite a negative SARS-CoV-2 nucleocapsid IgG test that was used to exclude those with a prior infection.

Additional limitations of our study include that breakthrough infections occurred at different time points between 14 days after the second dose and sample collection. The temporal dynamics of the antibody response may have distorted the comparative analysis. Also, the small sample size of participants with breakthrough infection further compromised the validity of this study group.

In summary, the results of our study demonstrate age-dependent waning of the humoral response with recoverability after breakthrough-infection in the elderly living in LTCFs. Therefore, booster vaccination ought to be considered, especially in this vulnerable group of patients (Interim statement on booster doses for COVID-19 vaccination, 2021). Since we did not discover a correlation between time passed since the second dose and anti-RBD IgG within the timeframe of the study, a booster vaccine earlier than 5 months after vaccination may be considered.

The fact that also the majority of HCWs did not have detectable neutralizing antibodies against the Delta variant highlights the importance of upholding the immune response independently of older age. An increase of efficacy of booster vaccination throughout different age groups has recently been documented (Arbel et al., 2021; Bar-On et al., 2021).

## Data Availability

The original contributions presented in the study are included in the article and Supplementary Material, further inquiries can be directed to the corresponding author.
