# Supplementary material for: Low But Recoverable Markers of Humoral Immune Response to BNT162b2 in Elderly LTCF Residents Five to Seven Months After Two-Dose Vaccination
Source: Front Aging. 2022 Apr 25;3:883724. doi: 10.3389/fragi.2022.883724 (PMC9261435; doi:10.3389/fragi.2022.883724)
Supplement: Supplementary file 1 [file DataSheet1.docx]

## **Supplementary Table 1**: Detailed listing of the **Charlson Comorbidity Index (CCI)** by study group. Percentages were calculated as indicated within the brackets. Wilcoxon rank sum test was used to test for homogeneity between study groups. The p-values can be found in column 6 respectively.

| Disease group | Weight | Group 1 (n = 298)  *SARS-CoV-2 naïve residents* | Group 2 (n = 114)  *SARS-CoV-2 naïve residents* | Group 3 (n = 14)  *Residents with breakthrough infection* | p-values  Group 1 vs. Group 2  Group 1 vs. Group 3 |
| --- | --- | --- | --- | --- | --- |
| Age Score | 0: < 50  1: ≥ 50  2: ≥ 60  3: ≥ 70  4: ≥ 80 | 0: 0% (0/298)  1: 0% (0/298)  2: 0% (0/298)  3: 14.8% (44/298)  4: 85.2% (254/298) | 0: 34.2% (39/114)  1: 40.3% (46/114)  2: 24.6% (28/114)  3: 0.9% (1/114)  4: 0% (0/114) | 0: 0% (0/14)  1: 0% (0/14)  2: 0% (0/14)  3: 0% (0/14)  4: 100% (14/14) | p_1 vs. 2_ < 0.001  p_1 vs. 3_ = 0.121 |
| Myocardial infarction | 1 | 7.2% (21/293) | 0% (0/114) | 14.3% (2/14) | p_1 vs. 2_ = 0.003  p_1 vs. 3_ = 0.324 |
| Congestive heart failure | 1 | 22.5% (66/293) | 1.8% (2/114) | 21.4% (3/14) | p_1 vs. 2_ < 0.001  p_1 vs. 3_ = 0.924 |
| Peripheral vascular disease | 1 | 7.9% (23/293) | 0% (0/114) | 14.3% (2/14) | p_1 vs. 2_ = 0.002  p_1 vs. 3_ = 0.391 |
| Cerebrovascular disease | 1 | 21.8% (64/293) | 0% (0/114) | 21.4% (3/14) | p_1 vs. 2_ < 0.001  p_1 vs. 3_ = 0.971 |
| Chronic obstructive pulmonary disease | 1 | 7.2% (21/293) | 3.5% (4/114) | 0% (0/14) | p_1 vs. 2_ = 0.168  p_1 vs. 3_ = 0.300 |
| Other chronic pulmonary disease | 1 | 3.8% (11/293) | 3.5% (4/114) | 0% (0/14) | p_1 vs. 2_ = 0.906  p_1 vs. 3_ = 0.461 |
| Rheumatic disease | 1 | 5.5% (16/293) | 1.8% (2/114) | 7.1% (1/14) | p_1 vs. 2_ = 0.103  p_1 vs. 3_ = 0.788 |
| Dementia | 1 | 45.4% (133/293) | 0% (0/114) | 64.3% (9/14) | p_1 vs. 2_ < 0.001  p_1 vs. 3_ = 0.167 |
| Hemiplegia, tetraplegia | 2 | 2.0% (6/293) | 0% (0/114) | 0% (0/14) | p_1 vs. 2_ = 0.124  p_1 vs. 3_ = 0.589 |
| Diabetes | 1 | 22.5% (66/293) | 6.1% (7/114) | 21.4% (3/14) | p_1 vs. 2_ < 0.001  p_1 vs. 3_ = 0.924 |
| Diabetes with end organ damage | 2 | 2.7% (8/293) | 0% (0/114) | 0% (0/14) | p_1 vs. 2_ = 0.075  p_1 vs. 3_ = 0.532 |
| Moderate or severe kidney disease | 2 | 18.8% (55/293) | 0% (0/114) | 14.3% (2/14) | p_1 vs. 2_ < 0.001  p_1 vs. 3_ = 0.674 |
| Mild liver disease | 1 | 1.7% (5/293) | 0.9% (1/114) | 0% (0/14) | p_1 vs. 2_ = 0.534  p_1 vs. 3_ = 0.632 |
| Moderate or severe liver disease | 3 | 0.7% (2/293) | 0.9% (1/114) | 0% (0/14) | p_1 vs. 2_ = 0.842  p_1 vs. 3_ = 0.757 |
| (Peptic) Ulcer disease | 1 | 0.3% (1/293) | 0% (0/114) | 0% (0/14) | p_1 vs. 2_ = 0.533  p_1 vs. 3_ = 0.827 |
| Any malignancy including leukemia and lymphoma | 2 | 14.7% (43/293) | 2.6% (3/114) | 14.3% (2/14) | p_1 vs. 2_ < 0.001  p_1 vs. 3_ = 0.968 |
| Metastatic cancer | 6 | 0.3% (1/293) | 0% (0/114) | 7.1% (1/14) | p_1 vs. 2_ = 0.533  p_1 vs. 3_ = 0.002 |
| HIV/AIDS | 6 | 0% (0/293) | 0% (0/114) | 0% (0/14) | N.A. |
| CCI [median] | N.A. | **6**  IQR: 5 to 7  range: 3 to 14 | **1**  IQR: 0 to 2  range: 0 to 5 | **6**  IQR: 5 to 7.75  range: 4 to 12 | p_1 vs. 2_ < 0.001  p_1 vs. 3_ = 0.386 |

## **Supplementary Table 2**: Detailed listing of the **Drug Derived Complexity Index (DDCI)** by study group. Percentages were calculated as indicated within the brackets. Wilcoxon rank sum test was used to test for homogeneity between study groups. The p-values can be found in column 6 respectively.

| Substance group | Weight | Group 1 (n = 293)  *SARS-CoV-2 naïve residents* | Group 2 (n = 114)  *SARS-CoV-2 naïve HCWs* | Group 3 (n = 14)  *Residents with breakthrough infection* | p-values  Group 1 vs. Group 2  Group 1 vs. Group 3 |
| --- | --- | --- | --- | --- | --- |
| Antiarrhythmics | 1 | 0.3% (1/293) | 0% (0/114) | 0% (0/14) | p_1 vs. 2_ = 0.533  p_1 vs. 3_ = 0.827 |
| Immunosuppressants | -1 | 0.3% (1/293) | 1.8% (2/114) | 0% (0/14) | p_1 vs. 2_ = 0.135  p_1 vs. 3_ = 0.827 |
| Platelet aggregation inhibitors | 2 | 34.1% (100/293) | 4.4% (5/114) | 57.1% (8/14) | p_1 vs. 2_ < 0.001  p_1 vs. 3_ = 0.079 |
| Parenteral anticoagulants | 1 | 1.0% (3/293) | 0% (0/114) | 0% (0/14) | p_1 vs. 2_ = 0.279  p_1 vs. 3_ = 0.704 |
| Oral anticoagulants | 1 | 1.0% (3/293) | 0.9% (1/114) | 0% (0/14) | p_1 vs. 2_ = 0.893  p_1 vs. 3_ = 0.704 |
| Antineoplastic agents | 3 | 0.7% (2/293) | 0% (0/114) | 0% (0/14) | p_1 vs. 2_ = 0.377  p_1 vs. 3_ = 0.757 |
| Inhaled bronchodilators | 2 | 5.8% (17/293) | 4.4% (5/114) | 7.1% (1/14) | p_1 vs. 2_ = 0.571  p_1 vs. 3_ = 0.835 |
| Drugs for arterial hypertension | 1 | 78.2% (229/293) | 23.7% (27/114) | 78.6% (11/14) | p_1 vs. 2_ < 0.001  p_1 vs. 3_ = 0.971 |
| Antihyperglycemic therapy | 2 | 18.1% (53/293) | 6.1% (7/114) | 14.3% (2/14) | p_1 vs. 2_ = 0.002  p_1 vs. 3_ = 0.717 |
| Drugs for hypertensive heart disease | 3 | 36.9% (108/293) | 4.4% (5/114) | 35.7% (5/14) | p_1 vs. 2_ < 0.001  p_1 vs. 3_ = 0.931 |
| Drugs for acid related diseases | 1 | 23.9% (70/293) | 2.6% (3/114) | 35.7% (5/14) | p_1 vs. 2_ < 0.001  p_1 vs. 3_ = 0.315 |
| Lipid modifying agents | -2 | 37.9% (111/293) | 5.3% (6/114) | 64.3% (9/14) | p_1 vs. 2_ < 0.001  p_1 vs. 3_ = 0.048 |
| Systemic corticosteroids | 2 | 4.4% (13/293) | 0% (0/114) | 14.3% (2/14) | p_1 vs. 2_ = 0.022  p_1 vs. 3_ = 0.096 |
| Opioids | 6 | 16.7% (49/293) | 0% (0/114) | 28.6% (4/14) | p_1 vs. 2_ < 0.001  p_1 vs. 3_ = 0.253 |
| Anti-Parkinson drugs | 4 | 11.6% (34/293) | 0% (0/114) | 21.4% (3/14) | p_1 vs. 2_ < 0.001  p_1 vs. 3_ = 0.271 |
| Antipsychotics | 3 | 28.0% (82/293) | 0% (0/114) | 57.1% (8/14) | p_1 vs. 2_ < 0.001  p_1 vs. 3_ = 0.019 |
| Anti-dementia drugs | 4 | 10.2% (30/293) | 0% (0/114) | 21.4% (3/14) | p_1 vs. 2_ < 0.001  p_1 vs. 3_ = 0.187 |
| Total number of prescription drugs [median] | N.A. | **7**  IQR: 5 to 8  range: 0 to 16 | **0.5**  IQR: 0 to 1  range: 0 to 5 | **7**  IQR: 5 to 8  range: 2 to 15 | p_1 vs. 2_ < 0.001  p_1 vs. 3_ = 0.002 |
| DDCI [median] | N.A. | **5**  IQR: 2 to 8  range: -2 to 20 | **0**  IQR: 0 to 1  range: -2 to 6 | **9.5**  IQR: 8.25 to 11.75  range: 0 to 16 | p_1 vs. 2_ < 0.001  p_1 vs. 3_ = 0.022 |
